# Cost-effectiveness and Benefit-to-Harm Ratio of Risk-Stratified Screening for Breast Cancer: A Life-Table Model

**DOI:** 10.1001/jamaoncol.2018.1901

**Published:** 2018-07-05

**Authors:** Nora Pashayan, Steve Morris, Fiona J. Gilbert, Paul D. P. Pharoah

**Affiliations:** 1 Department of Applied Health Research, University College London, London, England; 2 Department of Radiology, Cambridge Biomedical Campus, University of Cambridge, Cambridge, England; 3 Departments of Oncology and Public Health and Primary Care, Strangeways Research Laboratory, University of Cambridge, Cambridge, England

## Abstract

**Importance:**

The age-based or “one-size-fits-all” breast screening approach does not take into account the individual variation in risk. Mammography screening reduces death from breast cancer at the cost of overdiagnosis. Identifying risk-stratified screening strategies with a more favorable ratio of overdiagnoses to breast cancer deaths prevented would improve the quality of life of women and save resources.

**Objective:**

To assess the benefit-to-harm ratio and the cost-effectiveness of risk-stratified breast screening programs compared with a standard age-based screening program and no screening.

**Design, Setting, and Population:**

A life-table model was created of a hypothetical cohort of 364 500 women in the United Kingdom, aged 50 years, with follow-up to age 85 years, using (1) findings of the Independent UK Panel on Breast Cancer Screening and (2) risk distribution based on polygenic risk profile. The analysis was undertaken from the National Health Service perspective.

**Interventions:**

The modeled interventions were (1) no screening, (2) age-based screening (mammography screening every 3 years from age 50 to 69 years), and (3) risk-stratified screening (a proportion of women aged 50 years with a risk score greater than a threshold risk were offered screening every 3 years until age 69 years) considering each percentile of the risk distribution. All analyses took place between July 2016 and September 2017.

**Main Outcomes and Measures:**

Overdiagnoses, breast cancer deaths averted, quality-adjusted life-years (QALYs) gained, costs in British pounds, and net monetary benefit (NMB). Probabilistic sensitivity analyses were used to assess uncertainty around parameter estimates. Future costs and benefits were discounted at 3.5% per year.

**Results:**

The risk-stratified analysis of this life-table model included a hypothetical cohort of 364 500 women followed up from age 50 to 85 years. As the risk threshold was lowered, the incremental cost of the program increased linearly, compared with no screening, with no additional QALYs gained below 35th percentile risk threshold. Of the 3 screening scenarios, the risk-stratified scenario with risk threshold at the 70th percentile had the highest NMB, at a willingness to pay of £20 000 (US $26 800) per QALY gained, with a 72% probability of being cost-effective. Compared with age-based screening, risk-stratified screening at the 32nd percentile vs 70th percentile risk threshold would cost £20 066 (US $26 888) vs £537 985 (US $720 900) less, would have 26.7% vs 71.4% fewer overdiagnoses, and would avert 2.9% vs 9.6% fewer breast cancer deaths, respectively.

**Conclusions and Relevance:**

Not offering breast cancer screening to women at lower risk could improve the cost-effectiveness of the screening program, reduce overdiagnosis, and maintain the benefits of screening.

## Key Points

**Question:**

Can risk-stratified screening for breast cancer improve the cost-effectiveness and benefit-to-harm ratio of screening programs?

**Findings:**

In this cost-effectiveness study, a life-table model of a hypothetical cohort of 364 500 women finds that targeting screening to women at higher risk of breast cancer is associated with reduced overdiagnosis and reduced cost of screening without compromising quality-adjusted life-years gained and while maintaining reduced breast cancer deaths.

**Meaning:**

The cost-effectiveness and the benefit-to-harm ratio of breast screening programs could be improved by adopting a risk-stratified screening strategy.

## Teaser

This cost-effectiveness study uses a life-table model of a hypothetical cohort of 364 500 women to evaluate the cost-effectiveness and benefit to harm ratio of risk-stratified screening for breast cancer.

## Introduction

The Breast Cancer Screening Programme in the United Kingdom invites women in the general population aged 50 to 69 years for 2-view digital mammography every 3 years. However, the risk of developing breast cancer varies among women.^1^ This age-based or the “one-size-fits-all” screening approach does not take into account the individual variation in risk. To date, several studies in breast and prostate cancer have reported that tailoring screening to an individual’s risk level could improve the efficiency of the screening program and reduce its adverse consequences.^2,3,4,5,6,7^

Screening for breast cancer reduces deaths from the cancer.^8^ However, the trade-offs include overdiagnosis and overtreatment. Overdiagnosis is the detection by screening of tumors that would not have presented clinically in a person’s lifetime in the absence of screening. The Independent UK Panel on Breast Cancer Screening^8^ estimated that for every 10 000 women in the United Kingdom aged 50 years attending screening for the next 20 years, 56 deaths from breast cancer would be prevented, and 101 patients with breast cancer would be overdiagnosed. A cost-effectiveness analysis of the United Kingdom breast screening program, based on the findings of the Panel, showed that the program, compared with no screening, has 45% probability of being cost-effective at a threshold of £20 000 (US $26 800) per quality-adjusted life-year (QALY) gained.^9^

Genetic, lifestyle, and reproductive factors affect a woman’s risk for breast cancer. To date, genome-wide association studies have identified 310 breast cancer susceptibility loci^10^ that explain approximately 18% of the excess familial risk of breast cancer, with approximately 28% attributable to potentially all common variants in the genome.^10^ Assuming a log-additive model of interaction between loci, the known loci define a polygenic risk profile with a variance for the log relative risk distribution of 0.26. The estimated relative risks at the 1st and 99th percentiles are 0.22 and 3.55, respectively, compared with the average (population) risk. When the genetic susceptibility variants are combined with other epidemiological risk factors,^11^ the estimated relative risk at the 99th percentile increases to 4.67. Such a distribution could be used for risk stratification in screening programs at the population level.^4^

While the average 10-year absolute risk of breast cancer in women aged 50 years in the United Kingdom is 2.85%, women at the lowest and highest percentiles of the risk distribution have 0.53% and 9.96% 10-year risk, respectively (eFigure 1 in the Supplement). Offering screening to women at higher risk while sparing women whose risk is too low to justify the harms of screening may improve the benefit-to-harm ratio of screening. Risk-stratified screening would require assessing risk of all women, which would entail additional costs. However, these may be offset by eliminating repeated screening of women at lower risk and avoiding treatment of overdiagnosed cancers.

The aim of this study was to assess the benefit-to-harm ratio and cost-effectiveness of risk-stratified breast screening strategies that vary in risk threshold, using findings of the Independent UK Panel on Breast Cancer Screening and taking into account the uncertainty of the estimated benefits, harms, and costs.

## Methods

### Model Design

We used the life-table model that was developed to evaluate the cost-effectiveness of the National Health Service (NHS) Breast Screening Programme (NHSBSP)^9^ and extended it to account for risk-stratified screening. We simulated 3 hypothetical cohorts of 50-year-old women free of breast cancer followed up for 35 years. Each cohort consisted of 364 500 women, which is the 2009 population of 50-year-old women in England and Wales.^12^ The first cohort received no screening. The second cohort was offered breast screening mammography at age 50 years and every 3 years thereafter until age 69 years (ie, simulating the NHSBSP). And in the third cohort, risk estimation was carried out, and only the proportion of women in the population with a risk score greater than a threshold risk were offered screening every 3 years from age 50 years until age 69 years.

### Risk Distribution

For the base case model, we used a variance for the risk distribution of 0.43, which corresponds to both (1) the mid value between variance based on the known loci and on all the potential variants in the genome and (2) the combined variance of the known loci and epidemiological risk factors.^11^ Assuming a log-additive model of interaction between genetic and epidemiological risk factors,^13^ the distribution of risk on a relative risk scale is log-normal.^14^ The percentile rank associated with a given risk score (relative risk or age-conditional absolute risk) in the population or in cases can be calculated given the mean and variance of the log-normal relative risk distribution. We estimated (1) the proportion of the population that has a risk score greater than a given absolute risk threshold and (2) the proportion of cases that will occur within this high-risk subgroup. We calculated the relative risk associated with a risk score in the higher- and lower-risk subgroups considering truncated log-normal relative risk distribution.

### Input Parameters

We constructed a life table based on the predicted rates of age-specific incidence of breast cancer, breast cancer–specific mortality in the screened and unscreened population, and mortality from other causes among women with and without breast cancer. The estimation of the input parameters for the life-table models and the underlying assumptions have been described previously^9^ and are summarized in eTable 1 in the Supplement. We calculated the incidence and the cancer-specific mortality rates in the higher- and lower-risk groups by multiplying the overall predicted rates by the relative risk associated with a given risk score in the higher- and lower-risk subgroups. We applied the overdiagnosis estimate of the UK Independent Panel on Breast Cancer Screening to the number of cancers diagnosed in the higher-risk group (ie, in the screened group) during the screening period to calculate the number of overdiagnoses.

We modeled cost-effectiveness of age-based and risk-stratified screening compared with no screening from the NHS perspective using NHS costs for the screening program and treatment of breast cancer. We used an empirical estimate for the cost of risk assessment and literature-based estimates for the utility weights.

### Model Outputs

Model outputs included number of breast cancer diagnoses, number overdiagnosed, number of deaths from breast cancer, number of deaths from other causes, person-years of survival, QALYs, and total costs. The benefit-to-harm ratio was measured as the ratio of overdiagnoses to breast cancer deaths prevented.

### Sensitivity Analysis

We studied 99 scenarios of risk-stratified screening strategies corresponding to each percentile risk score. In univariate deterministic sensitivity analysis, we varied the cost of risk estimation, adherence to screening recommendation, the variance of the risk distribution, and baseline incidence of breast cancer and examined the effects on the study outcomes. To account for the uncertainty in the estimated input parameters, we ran probabilistic sensitivity analyses by recalculating the output of the model after sampling independently each parameter from an underlying probabilistic distribution (eTable 1 in the Supplement). We recalculated the model 2000 times for each of the screening strategies: no screening, age-based screening, and risk-stratified screening.

### Cost-effectiveness Analysis

We calculated the incremental cost-effectiveness ratio (ICER) as the difference in mean costs (based on the 2000 simulations) between the screened and unscreened cohorts divided by the difference in mean QALYs between the 2 cohorts. We calculated the net monetary benefit (NMB) for no screening, age-based screening, and for the 99 scenarios of risk-stratified screening as the mean QALYs multiplied by a given willingness to pay (WTP) for a QALY, minus the total cost. The screening strategy with the highest NMB for a given WTP was considered the most cost-effective. We calculated incremental NMBs (screening vs no screening) to generate cost-effectiveness acceptability curves, which are a summary of the proportion of times the incremental NMB is positive, ie, that the screening strategy is cost-effective compared with no screening, for a given WTP for a QALY. All future costs and health outcomes were discounted at a rate of 3.5%.^15^ The analysis was performed using STATA/SE version 14.0.

## Results

### Benefits and Harms of Screening

eFigure 2 in the Supplement compares the model-based estimate of age-specific breast cancer incidence to the observed age-specific incidence for 2009. eTable 2 in the Supplement details the mean of the key outputs under the base case scenario for the screened (age-based and risk-based) and the unscreened cohorts, following 2000 simulations and discounting at 3.5%.

**Figure 1.**
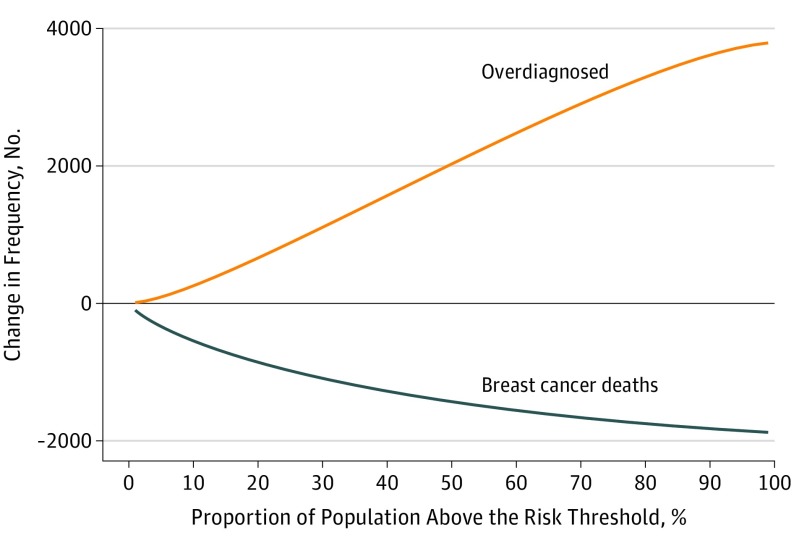
Change in Number of Overdiagnoses and Breast Cancer Deaths Averted in Risk-Stratified Screening Compared With No Screening The proportion of the population above the risk threshold corresponds to 100 minus the percentile risk. For example, 30% of the population above the risk threshold corresponds to 70th percentile of the risk distribution.

Among the 364 500 hypothetical women aged 50 years followed up to age 85 years, there were 1913 (95% CI, 842-2714) fewer deaths from breast cancer and 3819 (95% CI, 2309-5291) overdiagnosed breast cancers in the age-based screened cohort than in the unscreened cohort. In the risk-based screening, as the risk threshold for screening increased, ie, a lower proportion of the population screened, the number of overdiagnosed breast cancers and the number of breast cancer deaths prevented decreased. The ratio of overdiagnosis to cancer death prevented increased from 0.07 at the 99th percentile of the risk distribution (ie, 1% of the population with risk above the risk threshold and screened) to 0.99 at the 71st percentile and to 2.01 at the 1st percentile (Figure 1). There were more overdiagnosed cases than breast cancer deaths prevented when screening was targeted to women at a risk threshold of 70th percentile or less.

### Cost-effectiveness

**Figure 2.**
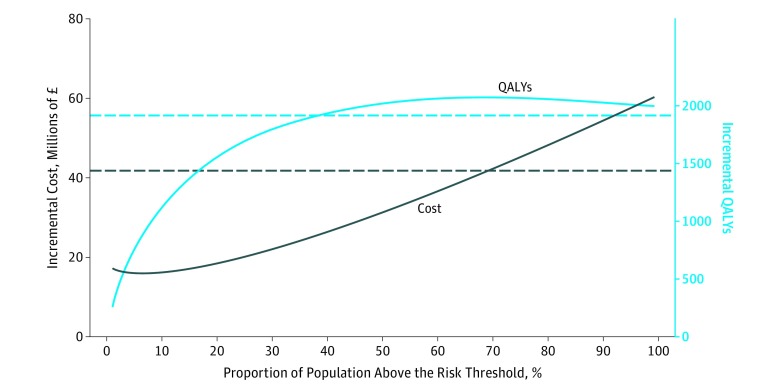
Incremental Cost and Incremental Quality-Adjusted Life-Years (QALYs) of Risk-Stratified Screening Compared With No Screening Dashed lines correspond to the incremental cost and incremental QALYs of age-based screening compared with no screening. The proportion of the population above the risk threshold corresponds to 100 minus the percentile risk. For example, 30% of the population above the risk threshold corresponds to 70th percentile of the risk distribution. To convert pounds sterling to US $, multiply by 1.34.

Compared with no screening, age-based screening was associated with an additional 1916 QALYs (95% CI, 2073-6073) at an additional cost of £41.9 M (95% CI, £41.7 million to £69.3 million) (to convert to US $, multiply by 1.34), giving an incremental cost-effectiveness ratio of £21 854 per QALY gained. In the risk-based screening, compared with no screening, the incremental cost increased linearly from £17.2 million to £60.2 million as the percentile risk threshold was lowered, while the incremental QALY increased from 258 to 2000 almost reaching a plateau by the 35th percentile of the risk distribution (Figure 2). The ICER at the 1st percentile of the risk distribution was £30 107 per QALY gained, and this declined when the risk threshold increased, with a minimum value of £11 911 per QALY at the 77th percentile, then increasing to £66 445 per QALY at the 99th percentile (eFigure 3 in the Supplement).

### Net Monetary Benefit

eFigure 4 in the Supplement shows the probability of risk-based screening being cost-effective at WTP thresholds of £20 000 and £30 000 per QALY gained. At the £20 000 threshold, targeting screening to women at the 71st or 70th percentile of the risk distribution was the most cost-effective strategy with a 72% probability of being cost-effective. eFigure 5 in the Supplement presents the cost-effectiveness planes related to each risk-stratified screening scenario; eFigure 6 in the Supplement presents the cost-effectiveness acceptability curves for each risk-stratified screening scenario; and eFigure 7 in the Supplement presents the probability of each scenario being cost-effective at a WTP of £20 000 per QALY.

### Sensitivity Analysis

eFigure 8A-D in the Supplement shows the outcomes of the univariate sensitivity analyses.

### Selection of the Optimal Screening Strategies

The Table details the key outcomes per year of 5 risk-stratified screening scenarios for 10 000 women compared with age-based screening, where the risk cutoff was set at the 10th, 25th, 32nd, 62nd, and 70th percentiles. The 10th percentile risk cutoff scenario corresponded to a very low–risk group (less than 1% 10-year absolute risk); the 25th percentile, ICER of risk-based screening lower than ICER of age-based screening (1.48% 10-year risk); the 32nd percentile, ICER lower than £20 000 per QALY (1.69% 10-year risk); the 62nd percentile, incremental QALYs more than that of age-based screening (2.81% 10-year risk); and the 70th percentile, the highest NMB at a WTP threshold of £20 000 per QALY gained (3.24% 10-year risk). At the 70th percentile risk threshold, the program would cost £537 985 less, yield 443 more QALYs, have 75 (71.4%) fewer overdiagnoses, and avert 23 (9.6%) fewer breast cancer deaths, compared with age-based screening. In contrast, at the 32nd percentile risk threshold, the program would cost £20 066 less, yield 450 more QALYs, have 27 (26.7%) fewer overdiagnoses, and avert 7 (2.9%) fewer breast cancer deaths.

**Table.** Differences in Outcomes per Year of Risk-Based Screening Compared With Age-based Screening Among 10 000 Women Screened From Age 50-69 Years and Followed up to Age 85 Years

Screening Strategy	Cases, No.			QALYs	Cost, £^b^
	Breast Cancer	Overdiagnosed	Breast Cancer Deaths		
Age-based screening	875	105	239	128 892	5 634 182
Risk-stratified screening^a^					
10th percentile	859	98	241	129 341	5 979 653
Difference vs age-based screening	−16	−7	+2	+449	+345 471
25th percentile	843	85	244	129 342	5 726 033
Difference vs age-based screening	−32	−20	+5	+450	+91 851
32nd percentile	834	77	246	129 342	5 614 116
Difference vs age-based screening	−41	−28	+7	+450	−20 066
62nd percentile	790	40	257	129 338	5 189 158
Difference vs age-based screening	−85	−65	+18	+446	−445 024
70th percentile	776	30	262	129 335	5 096 197
Difference vs age-based screening	−99	−75	+23	+443	−537 985

Abbreviation: QALYs, quality-adjusted life-years.

^a^ Percentile risk categories are reported from 0 risk. For example, the 10th percentile indicates that 10% of the population is within the risk category and 90% of the population is above the risk threshold. The 10-year absolute risk equivalent for the 10th, 25th 32nd, 68th and 70th percentiles of risk distribution are 0.99%, 1.48%, 1.69%, 2.81%, and 3.24%, respectively.

^b^ To convert to US $, multiply by 1.34.

## Discussion

A risk-stratified screening strategy could improve the benefit-to-harm ratio and the cost-effectiveness of the breast screening program. The relationship between the cost of the program and the QALYs gained shows diminishing return with offering screening to women at lower risk. The lower the risk threshold, ie, the larger the proportion of women offered screening, the higher would be the cost of the program, while the gain in QALYs would flatten off after a certain risk threshold. Lowering the risk threshold for screening would increase overdiagnosis to a greater extent than it would reduce breast cancer deaths.

*The European Guide on Quality Improvement in Comprehensive Cancer Control*^16^ recommends quantitative estimation of the benefits, harms, and cost-effectiveness of a screening program to decide on implementation. The National Institute for Health and Care Excellence^17^ recommends a cost-effectiveness threshold of £20 000 per QALY gained. However, there is no threshold for benefit-to-harm ratio. Of the cost-effective risk-stratified screening strategies, the optimal strategy would depend on the harm-benefit trade-offs deemed acceptable.

There are different approaches to risk-stratified screening. One approach would be to tailor screening modality, frequency, and start and stop age to an individual’s risk level. Other approaches include either (1) intensified screening for those at higher than a certain risk threshold, while those at lower risk receive the standard screening; or (2) offering no screening for those at lower risk, while those above the risk threshold receive the standard screening.^18^ We have modeled the second approach because of limited data available to model fully tailored screening. Yet it is not known how an individual’s risk relates to the biology and the natural history of the tumor or how these factors relate to the outcomes of screening. The interscreening interval depends on the *sojourn time*, ie, the preclinical screen-detectable period, and it is not known whether the sojourn time varies by risk level. Therefore, risk level currently provides limited guidance on how to vary the screening interval. Although much is known about the variation of mammographic sensitivity with breast cancer, it is not known how mammographic sensitivity compares between younger and older women at similarly higher risk. There are no direct estimates on the performance of supplemental screening modalities by risk and their effect on cancer specific mortality.

There are several studies that have evaluated the cost-effectiveness of tailoring the screening interval by breast cancer risk and mammographic density.^6,7,19,20^ The uncertainties in the key input parameters (detection rate, sensitivity, and mortality) due to lack of robust data made the findings of these studies only indicative.^20^ All of these studies suggest that risk-tailored screening could reduce harms and costs of screening.

We have estimated that for every 10 000 women aged 50 years who undergo age-based screening for the next 20 years in the United Kingdom, 52 deaths from breast cancer will be prevented, and 105 patients with breast cancer will be overdiagnosed. These data are comparable to the estimates of the Independent UK Panel on Breast Cancer Screening (56 breast cancer deaths and 101 overdiagnoses),^8^ which suggests that our model is reasonably robust.

Implementation of a risk-based screening program raises several challenges. These include (1) ensuring that genetic testing for stratification and eligibility for screening are acceptable to the public and the health care professionals; (2) preparing and training the workforce; (3) ensuring equitable access; and (4) having regulatory approvals.^18,21^ Evans and colleagues^22^ have found it is feasible to collect saliva for DNA extraction and genotyping of women attending the NHSBSP.^22^ Studies based on surveys suggest that women would accept undergoing genetic profiling to determine the frequency of screening.^23,24^ Yet no data exist on uptake of screening by risk group.

Unlike previously published studies of cost-effectiveness of risk-stratified screening, the present study modeled no screening for women at lower risk and standard screening for those at higher risk. In the setting of an established screening program, as in breast cancer, it may be more feasible to have a gradual introduction of risk-based screening by initially targeting screening to a subset of the population above a certain risk threshold. However, not offering screening to women at lower risk may not be acceptable^24^ because women have been encouraged to see screening as universally beneficial, and reduction in screening could be seen as service rationing.^25^ It is important to engage the public in decisions about screening program modification, to base the decision on robust evidence, and to communicate clearly the benefits and harms of screening.

### Limitations

These are model-based estimates that rely on assumptions. To minimize the assumptions and uncertainties associated with lack of data, we opted to develop a less complex model. We used a life-table modeling approach based on estimates from the Independent UK Panel on Breast Cancer Screening. We used overall cost of the NHSBSP, rather than unit costs of resources used, and utility decrement for cancer diagnosis regardless of cancer stage. Gray and colleagues^20^ have evaluated the cost-effectiveness of screening under the NHSBSP protocol compared with no screening using a decision analytic model taking into account the natural history of breast cancer and based on continuous time and tumor size growth model. They have used unit costs and utilities that vary by stage of breast cancer.^20^ Both analyses gave comparable ICERs (£21 854 and £23 197, respectively).

We have assumed that the probability of overdiagnosis does not vary by risk. This may not be the case. Studies in prostate cancer have shown that the probability of overdiagnosis is inversely associated with polygenic risk.^2,3^ There are no estimates yet on the association of overdiagnosis by risk in breast cancer. If increased risk is linked to increased risk of progression of the tumor, ie, shorter sojourn time, then overdiagnosis would be lower.^26^ However, this association is unlikely to substantially affect our estimates because the mean sojourn time of breast cancer is relatively short (2-4 years),^27,28^ and lower probabilities of overdiagnosis have been accounted for in the probabilistic sensitivity analysis.

## Conclusions

The cost-effectiveness and the benefit-to-harm ratio of the NHSBSP could be improved by adopting a risk-stratified screening strategy. The optimal risk threshold for risk-stratified screening depends on the acceptable trade-off between improving cost-effectiveness and maximizing benefits and minimizing harms of screening. Not offering screening to women in the lower tertile of the risk distribution would improve the cost-effectiveness of the breast screening program, reduce overdiagnosis while maintaining the benefits of screening. Robust data are needed to evaluate fully risk-tailored screening. Policy makers, health professionals, the public, and the scientific community have to work together to enable provision of screening program that can do more good than harm at an affordable cost.

## Notes

Supplement. **eTable 1.** Model input parameters **eTable 2.** Outcomes of three scenarios of screening – no screening, age-based screening, and risk-targeted screening **eFigure 1.** 10-year absolute risk of being diagnosed with breast cancer for women aged 50 years in England and Wales at each percentile of risk threshold. The variance of the risk distribution is 0.43 **eFigure 2.** Model based prediction of age-specific breast cancer incidence in population compared to observed age-specific incidence for 2009 **eFigure 3.** Incremental cost effectiveness ratios by risk-threshold (no screening is the comparator) **eFigure 4.** Net monetary benefits at willingness to pay (WTP) of £20,000 per QALY gained (a) and £30,000 per QALY gained (b) for no screening, age-based and risk-stratified screening scenarios **eFigure 5.** Cost-effectiveness planes of incremental cost vs. incremental QALYs of risk-stratified screening scenarios as compared to no screening. Results are based on 2,000 simulations **eFigure 6.** Cost-effectiveness acceptability curves for risk-stratified screening strategies for each percentile of risk threshold, considering willingness to pay (WTP) of £100 to £40,000 per QALY **eFigure 7.** The probability of each risk-stratified screening strategy of being cost-effective at willingness to pay (WTP) of £20,000 per QALY **eFigure 8A.** Deterministic sensitivity analysis - Incremental cost-effectiveness ratios by risk threshold considering breast cancer incidence rate where screening advances the diagnosis by 5 years, 7 years, and 9 years **eFigure 8B.** Deterministic sensitivity analysis - Incremental cost-effectiveness ratios by risk threshold considering 100%, 90% and 75% adherence to the screening recommendation for the higher and lower risk groups **eFigure 8C.** Deterministic sensitivity analysis - Incremental cost-effectiveness ratios by risk threshold considering cost of risk assessment of £25, £50, £75, and £100 **eFigure 8D.** Deterministic sensitivity analysis - Incremental cost-effectiveness ratios by risk threshold considering risk distribution variance of 0.28, 0.43, and 0.58 **eReferences.**
