# Supplementary material for: Cost-effectiveness and Benefit-to-Harm Ratio of Risk-Stratified Screening for Breast Cancer: A Life-Table Model
Source: JAMA Oncol. 2018 Jul 5;4(11):1504–10. doi: 10.1001/jamaoncol.2018.1901 (PMC6230256; doi:10.1001/jamaoncol.2018.1901)
Supplement: Supplement. — eTable 1. Model input parameters eTable 2. Outcomes of three scenarios of screening – no screening, age-based screening, and risk-targeted screening eFigure 1. 10-year absolute risk of being diagnosed with breast cancer for women aged 50 years in England and Wales at each percentile of risk threshold. The variance of the risk distribution is 0.43 eFigure 2. Model based prediction of age-specific breast cancer incidence in population compared to observed age-specific incidence for 2009 eFigure 3. Incremental cost effectiveness ratios by risk-threshold (no screening is the comparator) eFigure 4. Net monetary benefits at willingness to pay (WTP) of £20,000 per QALY gained (a) and £30,000 per QALY gained (b) for no screening, age-based and risk-stratified screening scenarios eFigure 5. Cost-effectiveness planes of incremental cost vs. incremental QALYs of risk-stratified screening scenarios as compared to no screening. Results are based on 2,000 simulations eFigure 6. Cost-effectiveness acceptability curves for risk-stratified screening strategies for each percentile of risk threshold, considering willingness to pay (WTP) of £100 to £40,000 per QALY eFigure 7. The probability of each risk-stratified screening strategy of being cost-effective at willingness to pay (WTP) of £20,000 per QALY eFigure 8A. Deterministic sensitivity analysis - Incremental cost-effectiveness ratios by risk threshold considering breast cancer incidence rate where screening advances the diagnosis by 5 years, 7 years, and 9 years eFigure 8B. Deterministic sensitivity analysis - Incremental cost-effectiveness ratios by risk threshold considering 100%, 90% and 75% adherence to the screening recommendation for the higher and lower risk groups eFigure 8C. Deterministic sensitivity analysis - Incremental cost-effectiveness ratios by risk threshold considering cost of risk assessment of £25, £50, £75, and £100 eFigure 8D. Deterministic sensitivity analysis - Incremental cost-effectiveness ratios by risk [file jamaoncol-4-1504-s001.pdf]

Supplementary Online Content

Pashayan N, Morris S, Gilbert FJ, Pharoah PDP. Cost-effectiveness and benefit-to-harm ratio of risk-stratified screening for breast cancer: a life-table model. *JAMA Oncology*. Published online July 5, 2018. doi:10.1001/jamaoncol.2018.1901

eTable 1. Model input parameters

eTable 2. Outcomes of three scenarios of screening – no screening, age-based screening, and risk-targeted screening

eFigure 1. 10-year absolute risk of being diagnosed with breast cancer for women aged 50 years in England and Wales at each percentile of risk threshold. The variance of the risk distribution is 0.43

eFigure 2. Model based prediction of age-specific breast cancer incidence in population compared to observed age-specific incidence for 2009

eFigure 3. Incremental cost effectiveness ratios by risk-threshold (no screening is the comparator)

eFigure 4. Net monetary benefits at willingness to pay (WTP) of £20,000 per QALY gained (a) and £30,000 per QALY gained (b) for no screening, age-based and risk-stratified screening scenarios

eFigure 5. Cost-effectiveness planes of incremental cost vs. incremental QALYs of risk-stratified screening scenarios as compared to no screening. Results are based on 2,000 simulations

eFigure 6. Cost-effectiveness acceptability curves for risk-stratified screening strategies for each percentile of risk threshold, considering willingness to pay (WTP) of £100 to £40,000 per QALY

eFigure 7. The probability of each risk-stratified screening strategy of being cost-effective at willingness to pay (WTP) of £20,000 per QALY

eFigure 8A. Deterministic sensitivity analysis - Incremental cost-effectiveness ratios by risk threshold considering breast cancer incidence rate where screening advances the diagnosis by 5 years, 7 years, and 9 years

© 2018 Pashayan N et al. *JAMA Oncology.*

eFigure 8B. Deterministic sensitivity analysis - Incremental cost-effectiveness ratios by risk threshold considering 100%, 90% and 75% adherence to the screening recommendation for the higher and lower risk groups

eFigure 8C. Deterministic sensitivity analysis - Incremental cost-effectiveness ratios by risk threshold considering cost of risk assessment of £25, £50, £75, and £100

eFigure 8D. Deterministic sensitivity analysis - Incremental cost-effectiveness ratios by risk threshold considering risk distribution variance of 0.28, 0.43, and 0.58

eReferences

This supplementary material has been provided by the authors to give readers additional information about their work.

© 2018 Pashayan N et al. *JAMA Oncology.*

Table 1: Model input parameters

Input parameter	Description (base-case value)	PSA Distribution (α, β) (mean, SD)*	Median [IQR]	eReferences
**Life table**				
Age-specific incidence of breast cancer in the absence of screening	• Expected one-year age band incidence in 2009 derived by multiplying the 1998 incidence by a factor of 1.45 to allow for the background increase in incidence over time			1;3
Age-specific incidence of screen-detected breast cancer	• Assuming that screening increases the incidence of breast cancer by both relative overdiagnosis and by advancing the diagnosis of breast cancer by seven years on average from age 50-69 and then incidence is dropped by ten per cent after screening stops<br>• Assuming that 75 per cent of women undergo screening<br>• These values were chosen empirically so that the predicted incidence approximates the observed population incidence for 2009			1
Age-specific mortality from breast cancer	• Derived from the breast cancer specific mortality rates in England and Wales, 2009<br>• Assuming these rates reflect weighted average of mortality rates in screened and unscreened cohorts in a population where 75 per cent had regular screening between ages 50 to 69<br>• Breast cancer mortality in screened = (observed breast mortality in the population * relative risk reduction) /(0.75*relative risk reduction+0.25)<br>• Breast cancer mortality in unscreened = (observed breast mortality in the population) / (0.75*relative risk reduction+0.25)			1;3
Age-specific mortality from other causes	• Derived from the mortality rates in England and Wales, 2009			1;3
Relative mortality reduction with screening	• Relative risk of breast cancer mortality associated with regular mammographic screening (0.8)<br>• Relative mortality risk reduction = 0.7+Log-Gamma(6,0.08)*0.25	Log-Gamma (6, 0.08)	0.81 [0.78,0.85]	1;2

© 2018 Pashayan N et al. *JAMA Oncology.*

Relative risk of death from non-breast cancer in women diagnosed with breast cancer	• Assuming the excess mortality with surgery is extremely small and adjuvant radiotherapy is associated with 12 per cent increase in mortality from other causes<br>• Based on cancer registration data, 50 per cent of breast cancer patients receive adjuvant radiotherapy<br>(1.06)<br>Relative risk of death=1+Log-Gamma(7,0.1)*0.1	Log-Gamma<br>(7, 0.1)	1.07 [1.05, 1.09]	1;4
Proportion overdiagnosed	• Proportion of the cancers diagnosed during the active screening period (19%)	Log-Normal<br>(0.174, 0.035)*	17.5% [15%, 20%]	1;2
**Utility**				
Health related utility	• Mean health related utility weight for a 50 year old woman without breast cancer (0.85)	Normal<br>(0.85, 0.01)*	0.85<br>[0.84, 0.86]	1
Annual decline in health related utility	• Annual decline in utility from age 50 to 80 (0.0043)	Normal<br>(0.0043, 0.001)*	0.0043 [0.0036, 0.005]	1;5
Relative reduction in quality of life associated with living after a diagnosis of breast cancer	• Mean health related utility decrement following diagnosis (0.9)<br>• Assuming the mean health-related utility decrement of 0.7, 0.8, and 0.9 in the three years following diagnosis and 0.95 thereafter compared to women of the same age<br>• Health related quality of life associated with diagnosis of breast cancer =0.85+0.167*Gamma(4,0.1); right truncated	Gamma<br>(4, 0.1)	0.91<br>[0.89, 0.94]	1;6
**Cost**				
Cost of the screening programme for each year of screening (Million)	• Estimated cost of the NHS breast screening programme £96 million<br>• Annual cost of the programme (£4.8 million)	Normal<br>(4.8, 0.8)*	4.8<br>[4.2, 5.3]	1;7
Cost of treating primary breast cancer	• If 72 per cent of cases detected by the NHSBSP were treated with local surgery and 27 per cent with mastectomy and 20 per cent of the patients also received radio therapy<br>• Average cost of treating primary breast cancer £1,450*0.72 + £2,810*0.27 + £1,800*0.20 (£2,163)<br>• Cost of treating primary cancer = 1800+Gamma(3,0.1)*1000	Gamma<br>(3, 0.1)	£2,066 [£1,971,<br>£2,186]	1;8
Cost of treating advanced	• Cost of treating metastatic breast cancer (£20,000)	Gamma	£20,658 [£19,714,	1;9

© 2018 Pashayan N et al. *JAMA Oncology*.

metastatic breast cancer	• Cost of treating metastatic cancer =18000+Gamma(3,0.1)*10000	(3, 0.1)	£21,864]	
Relative cost of treating a clinically detected patient with breast cancer compared with the cost if it had been detected earlier by screening	• Assuming clinically detected cancer to be more advanced than screen-detected cancer and the cost of treatment of the former would be higher<br>• Assuming 10 per cent increase in cost for treating cases in the unscreened group (1.1)	Normal<br><br>(1.1, 0.04)	1.10<br><br>[1.07, 1.13]	1
Cost of risk assessment	• Empirical estimate based on per variant research cost of genotyping (£50)<br>• Cost of risk assessment =Gamma(50,0.1)*10	Gamma<br><br>(50, 0.01)	£50<br><br>[£45, £55]	

PSA –Probabilistic sensitivity analysis
IQR – Interquartile range

© 2018 Pashayan N et al. *JAMA Oncology.*

eTable 2: Outcomes of three scenarios of screening – no screening, age-based screening, and risk-targeted screening.

Proportion at highest risk (%)	Breast cancer cases among the unscreened	Total breast cancer cases	Over-diag-nosed cases	Breast cancer deaths	Other causes deaths	PYRS	QALY	Cost (£)
**No screening**								
	26,826	26,826	-	10,631	239,113	6,421,926	4,696,181	163,486,827
**Age-based screening**								
	-	31,889	3,819	8,718	240,301	6,430,124	4,698,098	205,365,932
**Risk-based screening**								
1	25,604	26,761	7	10,505	239,180	6,422,481	4,712,717	180,862,880
2	24,725	26,772	21	10,436	239,218	6,422,780	4,712,873	180,373,280
3	23,959	26,798	39	10,378	239,250	6,423,035	4,712,999	180,073,328
4	23,263	26,834	61	10,326	239,280	6,423,265	4,713,107	179,886,912
5	22,617	26,875	85	10,279	239,307	6,423,475	4,713,202	179,780,480
6	22,010	26,919	112	10,234	239,333	6,423,671	4,713,288	179,735,312
7	21,435	26,967	141	10,192	239,357	6,423,855	4,713,366	179,739,472
8	20,887	27,017	171	10,153	239,380	6,424,030	4,713,438	179,784,720
9	20,361	27,069	204	10,115	239,402	6,424,197	4,713,504	179,865,152
10	19,856	27,122	237	10,078	239,424	6,424,356	4,713,566	179,976,176
11	19,369	27,177	272	10,044	239,444	6,424,509	4,713,623	180,114,256
12	18,898	27,232	308	10,010	239,464	6,424,656	4,713,676	180,276,544
13	18,442	27,289	345	9,978	239,484	6,424,798	4,713,727	180,460,672
14	17,999	27,346	384	9,946	239,503	6,424,936	4,713,774	180,664,704
15	17,569	27,404	423	9,916	239,521	6,425,068	4,713,818	180,887,008
16	17,150	27,462	463	9,886	239,539	6,425,198	4,713,860	181,126,144
17	16,743	27,521	503	9,857	239,556	6,425,323	4,713,899	181,380,912
18	16,345	27,580	545	9,829	239,573	6,425,445	4,713,937	181,650,224
19								

© 2018 Pashayan N et al. *JAMA Oncology.*

Proportion at highest risk (%)	Breast cancer cases among the unscreened	Total breast cancer cases	Over-diag-nosed cases	Breast cancer deaths	Other causes deaths	PYRS	QALY	Cost (£)
	15,957	27,640	587	9,802	239,590	6,425,563	4,713,972	181,933,152
20	15,578	27,699	630	9,776	239,606	6,425,678	4,714,005	182,228,864
21	15,207	27,759	673	9,750	239,622	6,425,791	4,714,037	182,536,608
22	14,844	27,820	716	9,724	239,637	6,425,901	4,714,067	182,855,744
23	14,490	27,880	761	9,700	239,653	6,426,008	4,714,095	183,185,664
24	14,142	27,940	805	9,675	239,668	6,426,112	4,714,122	183,525,792
25	13,802	28,001	850	9,652	239,682	6,426,215	4,714,147	183,875,648
26	13,468	28,061	896	9,628	239,697	6,426,315	4,714,172	184,234,800
27	13,141	28,122	941	9,606	239,711	6,426,412	4,714,195	184,602,800
28	12,820	28,182	987	9,584	239,725	6,426,508	4,714,216	184,979,296
29	12,505	28,242	1,034	9,562	239,738	6,426,602	4,714,237	185,363,936
30	12,196	28,303	1,080	9,540	239,752	6,426,694	4,714,257	185,756,384
31	11,892	28,363	1,127	9,519	239,765	6,426,784	4,714,275	186,156,320
32	11,594	28,423	1,173	9,499	239,778	6,426,872	4,714,293	186,563,504
33	11,301	28,483	1,220	9,479	239,790	6,426,958	4,714,310	186,977,632
34	11,014	28,543	1,268	9,459	239,803	6,427,043	4,714,326	187,398,496
35	10,731	28,603	1,315	9,439	239,815	6,427,126	4,714,341	187,825,840
36	10,453	28,662	1,362	9,420	239,827	6,427,208	4,714,355	188,259,488
37	10,180	28,721	1,409	9,401	239,839	6,427,288	4,714,368	188,699,200
38	9,911	28,780	1,457	9,383	239,851	6,427,366	4,714,381	189,144,816
39	9,647	28,839	1,504	9,365	239,863	6,427,443	4,714,393	189,596,128
40	9,387	28,898	1,552	9,347	239,874	6,427,519	4,714,404	190,053,008
41	9,132	28,956	1,599	9,330	239,885	6,427,593	4,714,415	190,515,280
42	8,880	29,015	1,646	9,312	239,896	6,427,666	4,714,425	190,982,768
43	8,633	29,072	1,694	9,296	239,907	6,427,738	4,714,435	191,455,376

© 2018 Pashayan N et al. *JAMA Oncology.*

Proportion at highest risk (%)	Breast cancer cases among the unscreened	Total breast cancer cases	Over-diag-nosed cases	Breast cancer deaths	Other causes deaths	PYRS	QALY	Cost (£)
44	8,390	29,130	1,741	9,279	239,918	6,427,808	4,714,444	191,932,960
45	8,150	29,187	1,788	9,263	239,928	6,427,877	4,714,452	192,415,360
46	7,915	29,244	1,835	9,247	239,938	6,427,945	4,714,460	192,902,480
47	7,683	29,301	1,882	9,231	239,949	6,428,011	4,714,467	193,394,240
48	7,455	29,357	1,929	9,215	239,959	6,428,077	4,714,474	193,890,480
49	7,230	29,413	1,975	9,200	239,969	6,428,141	4,714,480	194,391,120
50	7,010	29,469	2,022	9,185	239,978	6,428,204	4,714,486	194,896,064
51	6,792	29,525	2,068	9,170	239,988	6,428,267	4,714,492	195,405,216
52	6,578	29,580	2,114	9,156	239,997	6,428,327	4,714,497	195,918,480
53	6,368	29,634	2,160	9,142	240,007	6,428,387	4,714,501	196,435,792
54	6,161	29,689	2,206	9,128	240,016	6,428,446	4,714,506	196,957,072
55	5,957	29,742	2,251	9,114	240,025	6,428,504	4,714,509	197,482,208
56	5,756	29,796	2,296	9,100	240,034	6,428,561	4,714,513	198,011,168
57	5,559	29,849	2,341	9,087	240,042	6,428,617	4,714,516	198,543,872
58	5,365	29,902	2,386	9,074	240,051	6,428,671	4,714,519	199,080,272
59	5,174	29,954	2,430	9,061	240,059	6,428,725	4,714,522	199,620,272
60	4,986	30,006	2,474	9,049	240,068	6,428,778	4,714,524	200,163,840
61	4,801	30,058	2,518	9,036	240,076	6,428,830	4,714,526	200,710,896
62	4,619	30,109	2,561	9,024	240,084	6,428,881	4,714,527	201,261,440
63	4,441	30,159	2,604	9,012	240,092	6,428,931	4,714,529	201,815,360
64	4,265	30,209	2,647	9,000	240,100	6,428,980	4,714,530	202,372,640
65	4,092	30,259	2,689	8,989	240,107	6,429,028	4,714,531	202,933,232
66	3,923	30,308	2,731	8,978	240,115	6,429,075	4,714,531	203,497,104
67	3,756	30,357	2,772	8,966	240,122	6,429,122	4,714,532	204,064,224
68								

© 2018 Pashayan N et al. *JAMA Oncology.*

Proportion at highest risk (%)	Breast cancer cases among the unscreened	Total breast cancer cases	Over-diag-nosed cases	Breast cancer deaths	Other causes deaths	PYRS	QALY	Cost (£)
	3,592	30,405	2,814	8,956	240,130	6,429,167	4,714,532	204,634,544
69	3,431	30,453	2,854	8,945	240,137	6,429,212	4,714,532	205,208,016
70	3,273	30,501	2,894	8,934	240,144	6,429,256	4,714,532	205,784,624
71	3,118	30,547	2,934	8,924	240,151	6,429,298	4,714,531	206,364,352
72	2,966	30,594	2,974	8,914	240,158	6,429,340	4,714,530	206,947,168
73	2,817	30,640	3,012	8,904	240,164	6,429,381	4,714,529	207,533,024
74	2,670	30,685	3,051	8,895	240,171	6,429,421	4,714,528	208,121,952
75	2,527	30,729	3,089	8,885	240,177	6,429,460	4,714,527	208,713,904
76	2,386	30,774	3,126	8,876	240,183	6,429,499	4,714,526	209,308,864
77	2,248	30,817	3,163	8,867	240,189	6,429,537	4,714,524	209,906,848
78	2,113	30,860	3,199	8,858	240,196	6,429,573	4,714,523	210,507,824
79	1,981	30,903	3,235	8,850	240,201	6,429,609	4,714,521	211,111,808
80	1,852	30,945	3,270	8,841	240,207	6,429,644	4,714,519	211,718,784
81	1,726	30,986	3,304	8,833	240,213	6,429,678	4,714,517	212,328,784
82	1,603	31,027	3,338	8,825	240,218	6,429,711	4,714,514	212,941,808
83	1,483	31,067	3,371	8,817	240,224	6,429,744	4,714,512	213,557,856
84	1,366	31,106	3,404	8,810	240,229	6,429,774	4,714,509	214,176,976
85	1,252	31,145	3,436	8,802	240,234	6,429,805	4,714,507	214,799,184
86	1,141	31,183	3,467	8,795	240,239	6,429,835	4,714,504	215,424,544
87	1,033	31,220	3,497	8,788	240,244	6,429,863	4,714,501	216,053,056
88	928	31,257	3,527	8,782	240,248	6,429,891	4,714,498	216,684,816
89	827	31,293	3,556	8,775	240,253	6,429,918	4,714,495	217,319,904
90	729	31,328	3,584	8,769	240,257	6,429,943	4,714,492	217,958,368
91	635	31,362	3,611	8,763	240,261	6,429,968	4,714,489	218,600,352
92	545	31,396	3,637	8,758	240,265	6,429,991	4,714,485	219,245,984

© 2018 Pashayan N et al. *JAMA Oncology.*

Proportion at highest risk (%)	Breast cancer cases among the unscreened	Total breast cancer cases	Over-diag-nosed cases	Breast cancer deaths	Other causes deaths	PYRS	QALY	Cost (£)
93	458	31,428	3,662	8,753	240,269	6,430,013	4,714,482	219,895,440
94	375	31,460	3,686	8,748	240,273	6,430,034	4,714,478	220,548,928
95	297	31,491	3,709	8,743	240,276	6,430,054	4,714,474	221,206,768
96	224	31,520	3,730	8,738	240,279	6,430,073	4,714,471	221,869,344
97	156	31,549	3,750	8,735	240,282	6,430,089	4,714,466	222,537,232
98	94	31,575	3,768	8,731	240,285	6,430,104	4,714,462	223,211,392
99	40	31,601	3,784	8,728	240,287	6,430,116	4,714,458	223,893,568

PYRS – Person years of survival
QALY – Quality adjusted life years
Discounted at 3.5% per year
Average of 2000 simulations

© 2018 Pashayan N et al. *JAMA Oncology*.

eFigure 1: 10-year absolute risk of being diagnosed with breast cancer for women aged 50 years in England and Wales at each percentile of risk threshold. The variance of the risk distribution is 0.43.

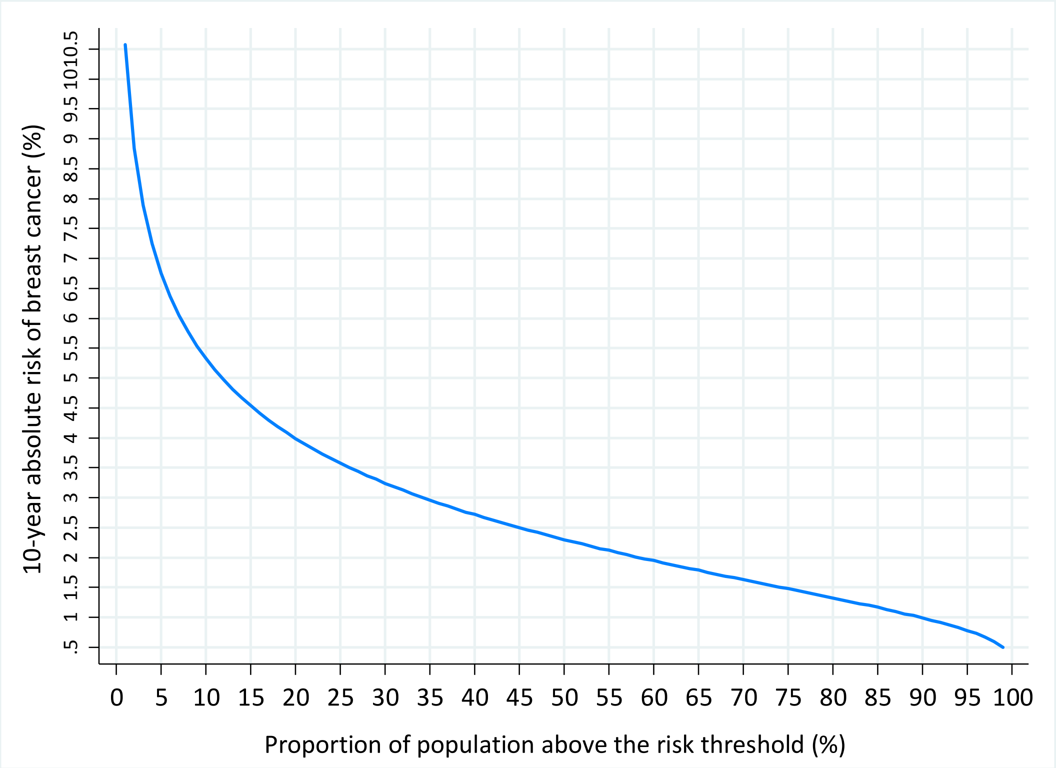

The population average 10-year risk is 2.85%.

© 2018 Pashayan N et al. *JAMA Oncology.*

eFigure 2: Model based prediction of age-specific breast cancer incidence in population compared to observed age-specific incidence for 2009.

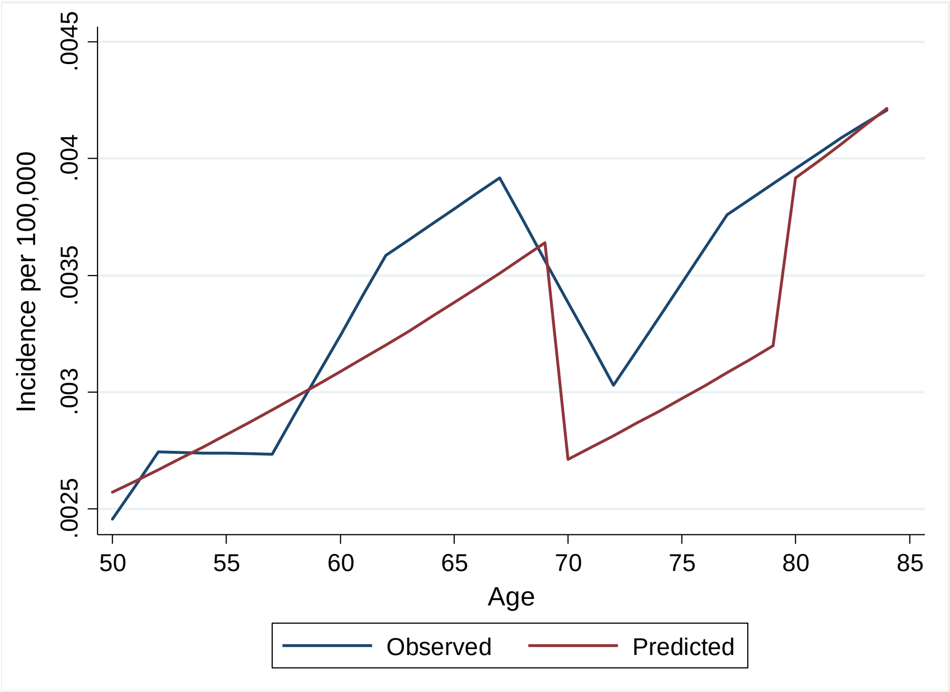

© 2018 Pashayan N et al. *JAMA Oncology*.

eFigure 3: Incremental cost effectiveness ratios by risk-threshold (no screening is the comparator)

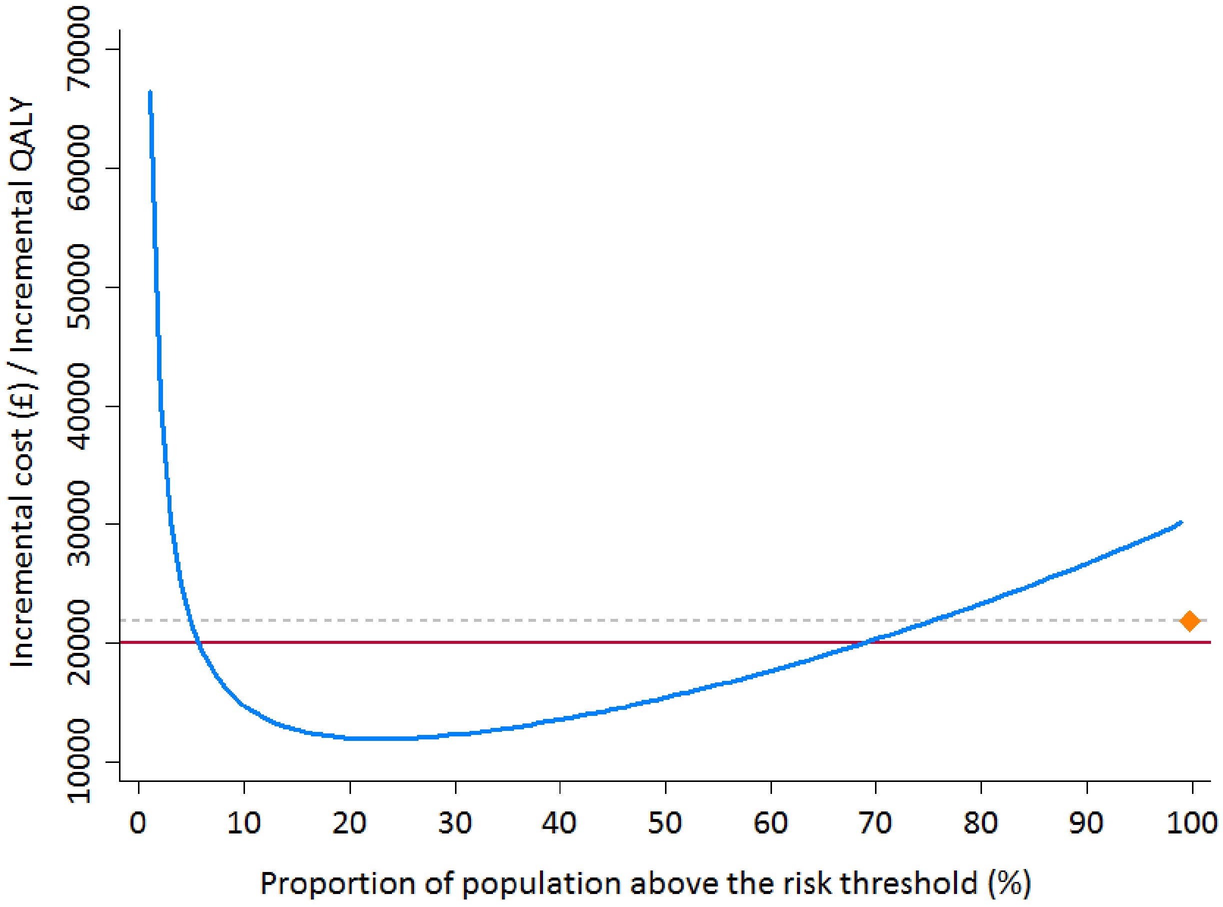

Red line: Threshold of £20,000 per QALY gained

Diamond: Incremental cost-effectiveness ratio for age-based screening

Incremental cost-effectiveness ratio is the difference in mean costs (based on 2,000 simulations) between the screened and unscreened cohorts divided by the difference in mean QALYs between the two cohorts.

© 2018 Pashayan N et al. *JAMA Oncology.*

eFigure 4. Net monetary benefits at willingness to pay (WTP) of £20,000 per QALY gained (a) and £30,000 per QALY gained (b) for no screening, age-based and risk-stratified screening scenarios.

a. **WTP £ 20,000 per QALY**

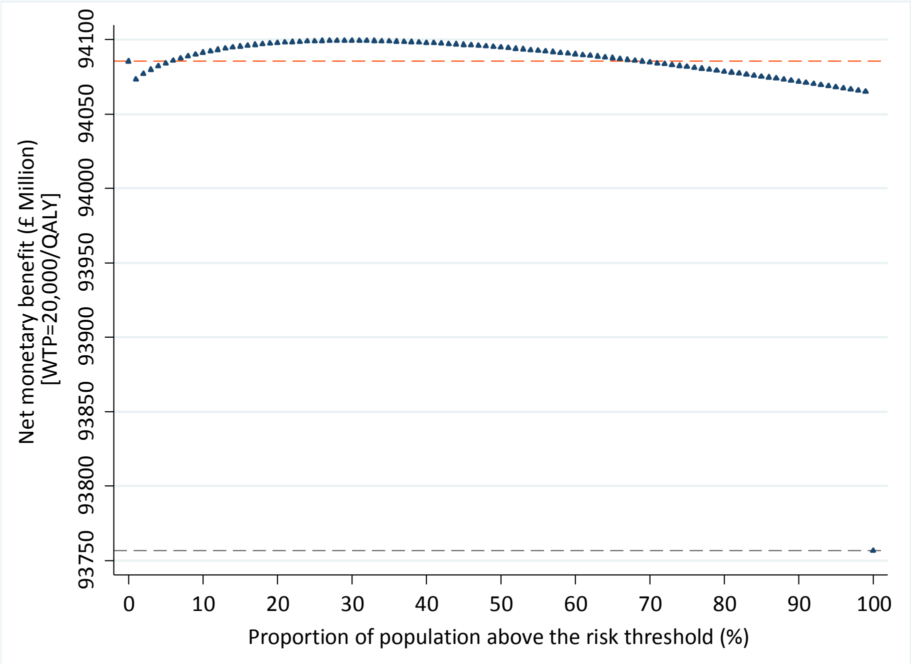

b. **WTP £30,000 per QALY**

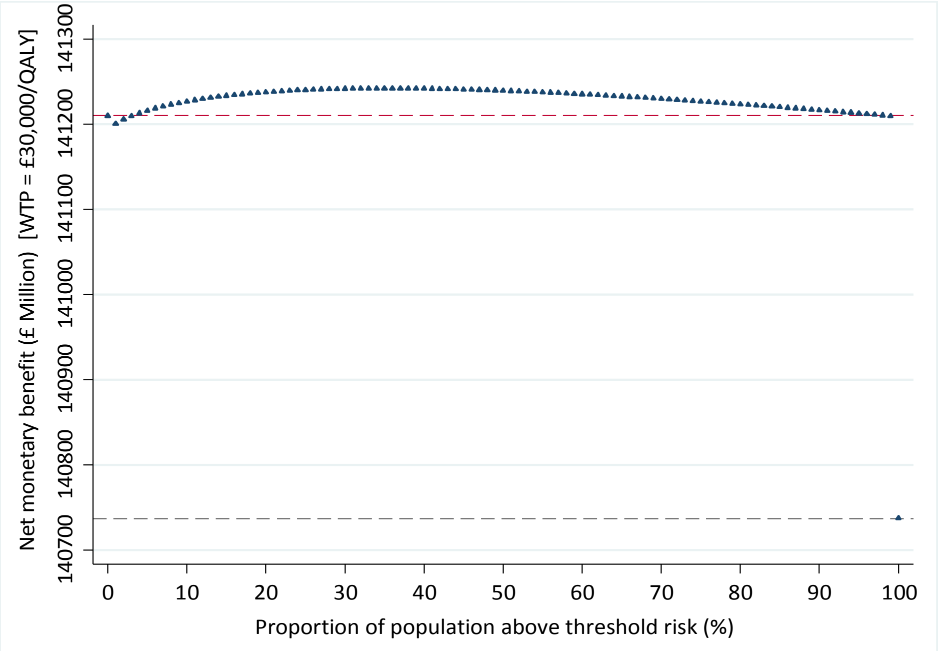

Red dashed line indicates the NMB of no screening
Grey dashed line indicates the NMB of age-based screening
The highest NMB value corresponds to the most-cost-effective scenario: in a. targeting screening to 30% women at highest risk and in b. to 35% of women at highest risk.

© 2018 Pashayan N et al. *JAMA Oncology.*

eFigure 5: Cost-effectiveness planes of incremental cost vs. incremental QALYs of risk-stratified screening scenarios as compared to no screening. Results are based on 2,000 simulations.

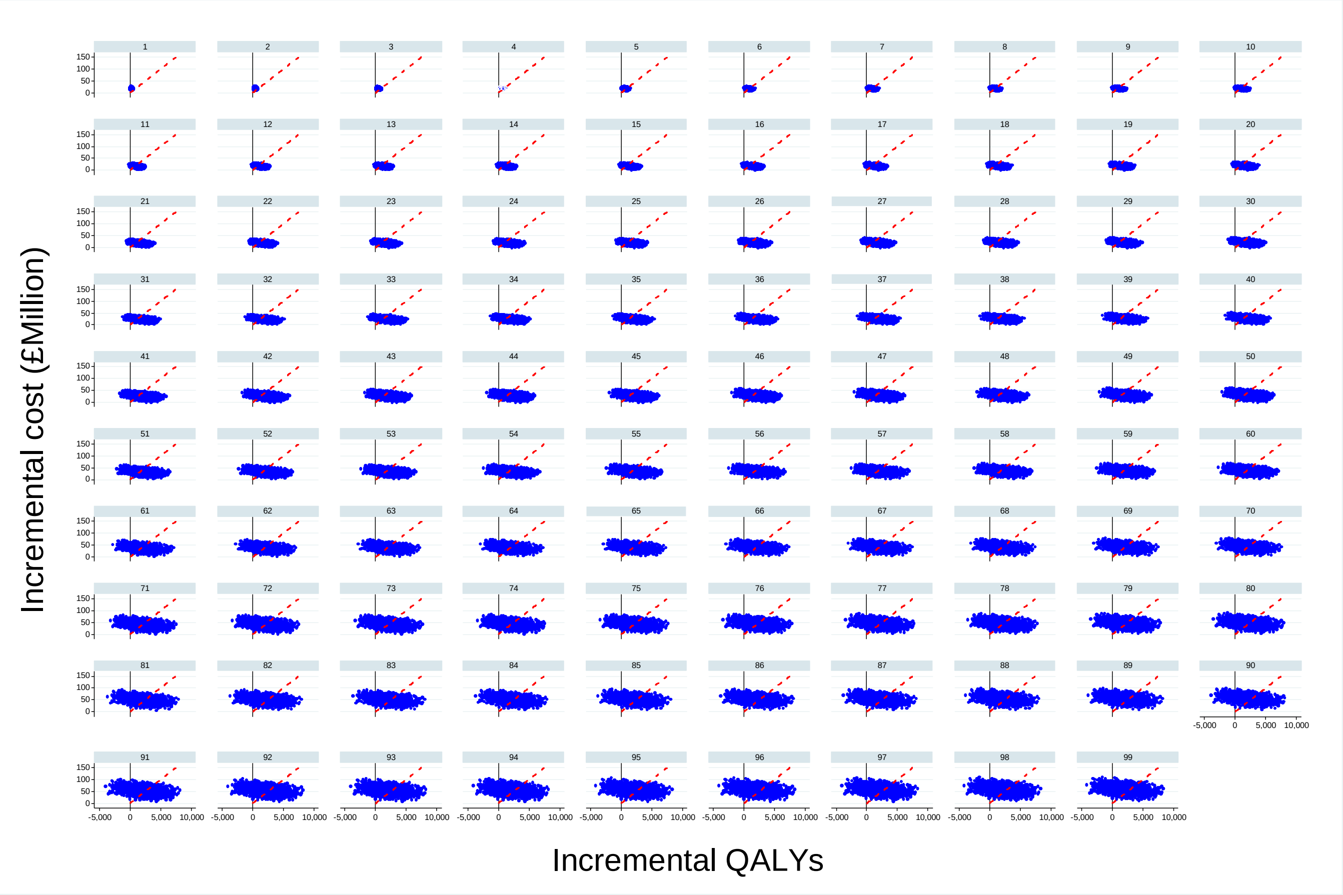

Each graph represents percentile risk threshold for risk-stratified screening strategy. The first graph is for 99th percentile risk threshold and the last graph for the 1st percentile.

© 2018 Pashayan N et al. *JAMA Oncology*.

eFigure 6: Cost-effectiveness acceptability curves for risk-stratified screening strategies for each percentile of risk threshold, considering willingness to pay (WTP) of 100 to 40,000 per QALY.

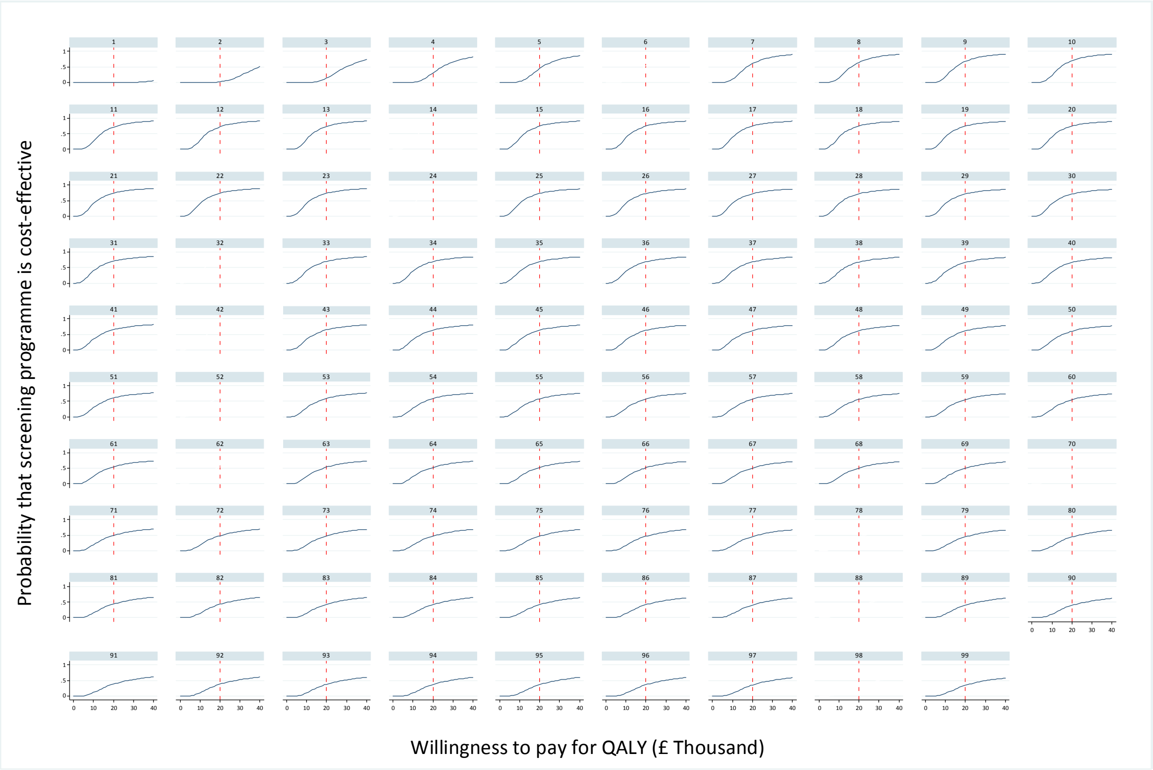

Each graph represents percentile risk threshold for risk-stratified screening strategy. The first graph is for 99th percentile risk threshold and the last graph for the 1st percentile.

The red line represents WTP of 20,000 per QALY.

The analysis is based on net monetary benefit (NMB) approach using 2,000 simulations.

© 2018 Pashayan N et al. *JAMA Oncology.*

eFigure 7: The probability of each risk-stratified screening strategy of being cost-effective at willingness to pay (WTP) of £20,000 per QALY.

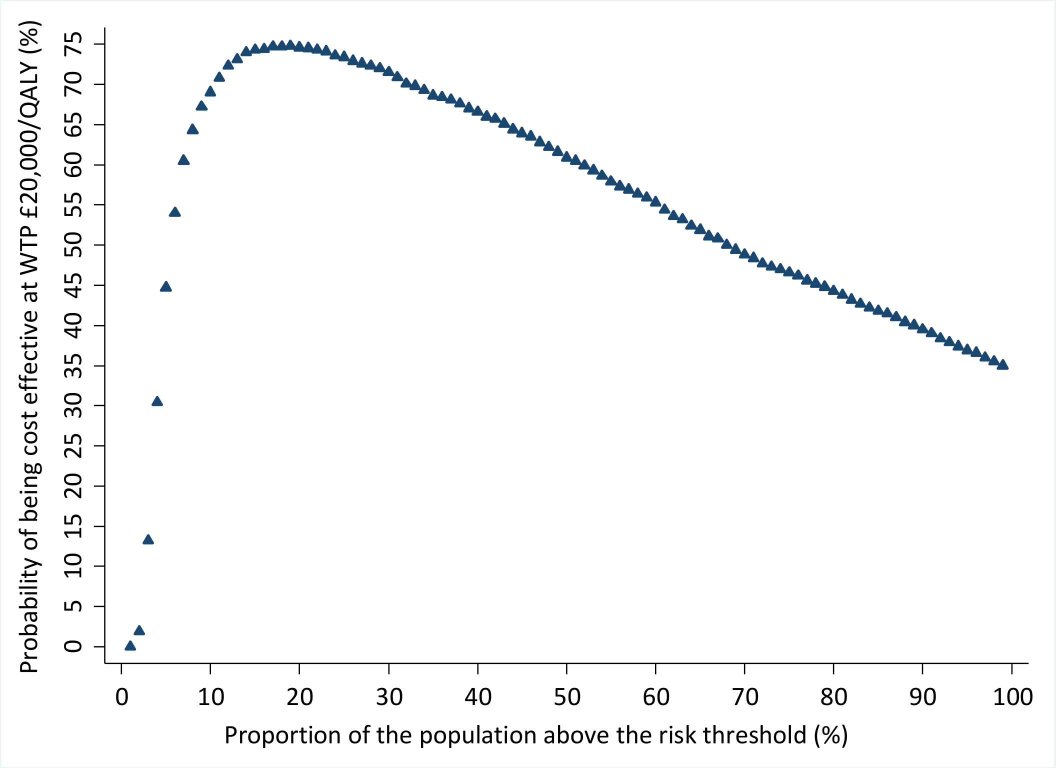

The analysis is based on net monetary benefit (NMB) approach using 2,000 simulations.

© 2018 Pashayan N et al. *JAMA Oncology.*

eFigure 8A: Deterministic sensitivity analyses –

a. **Incremental cost-effectiveness ratios by risk threshold considering breast cancer incidence rate where screening advances the diagnosis by 5 years, 7 years, and 9 years**

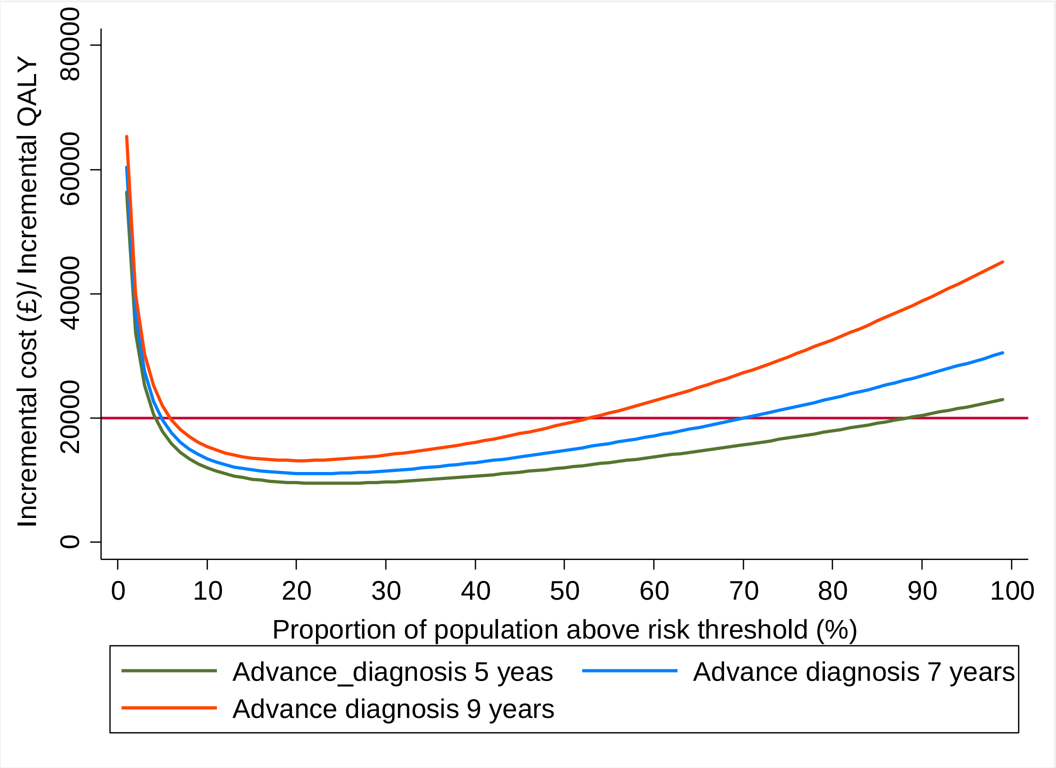

Red line: Threshold of £20,000 per QALY gained

© 2018 Pashayan N et al. *JAMA Oncology.*

b. eFigure 8B. Incremental cost-effectiveness ratios by risk threshold considering 100%, 90% and 75% adherence to the screening recommendation for the higher and lower risk groups.

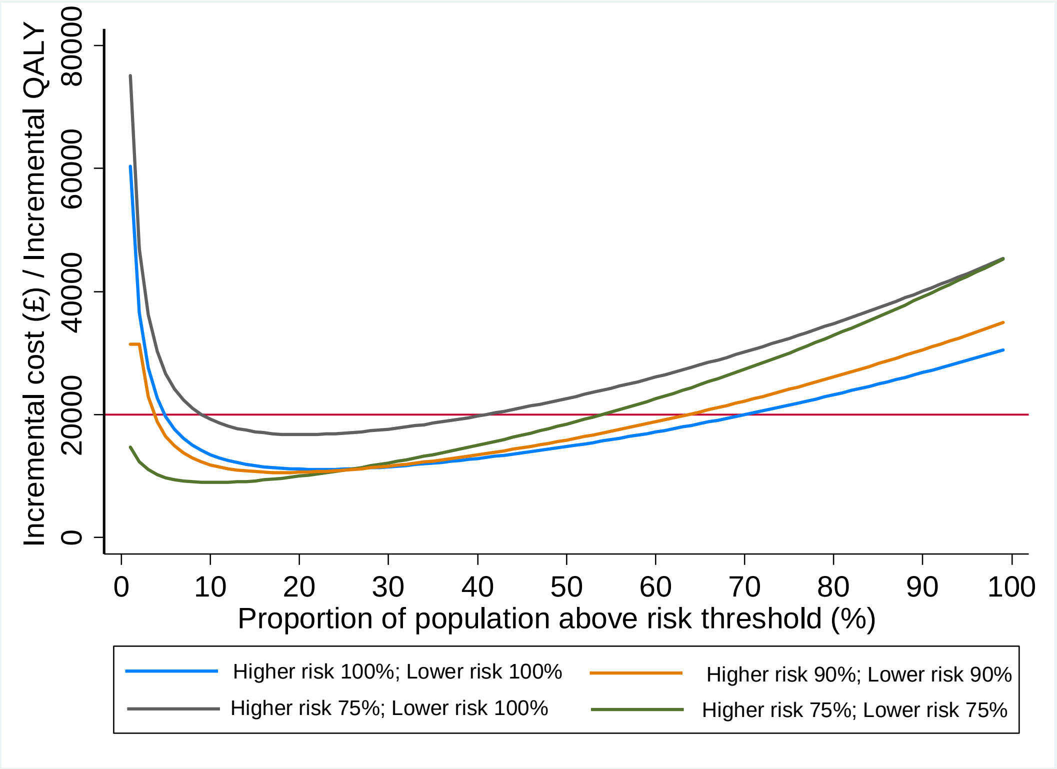

Red line: Threshold of £20,000 per QALY gained

© 2018 Pashayan N et al. *JAMA Oncology.*

c. **eFigure 8C. Incremental cost-effectiveness ratios by risk threshold considering cost of risk assessment of £25, £50, £75, and £100.**

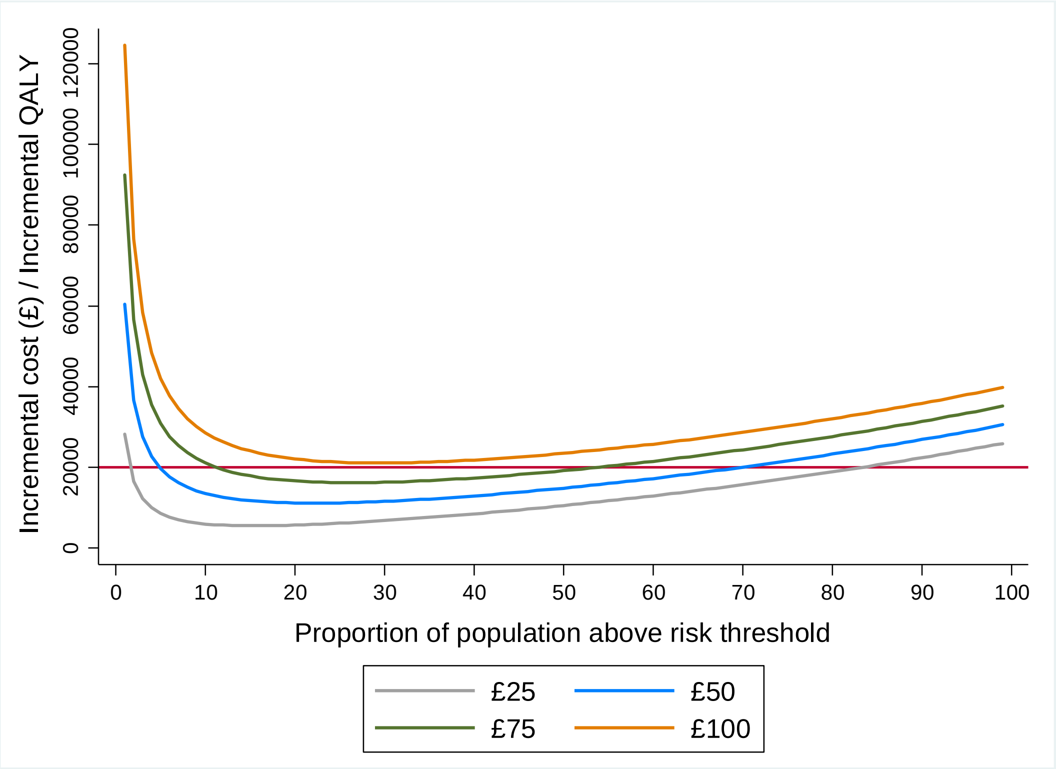

Red line: Threshold of £20,000 per QALY gained
Risk is based on polygenic profiling of the breast cancer germline genetic susceptibility variants and questionnaire based epidemiological risk factors (age at menarche, parity, age at first birth, combined menopausal hormone therapy, body mass index, benign breast disease, alcohol intake, smoking, and family history of breast cancer in first-degree relatives)

© 2018 Pashayan N et al. *JAMA Oncology.*

d. eFigure 8D. Incremental cost-effectiveness ratios by risk threshold considering risk distribution variance of 0.28, 0.43, and 0.58.

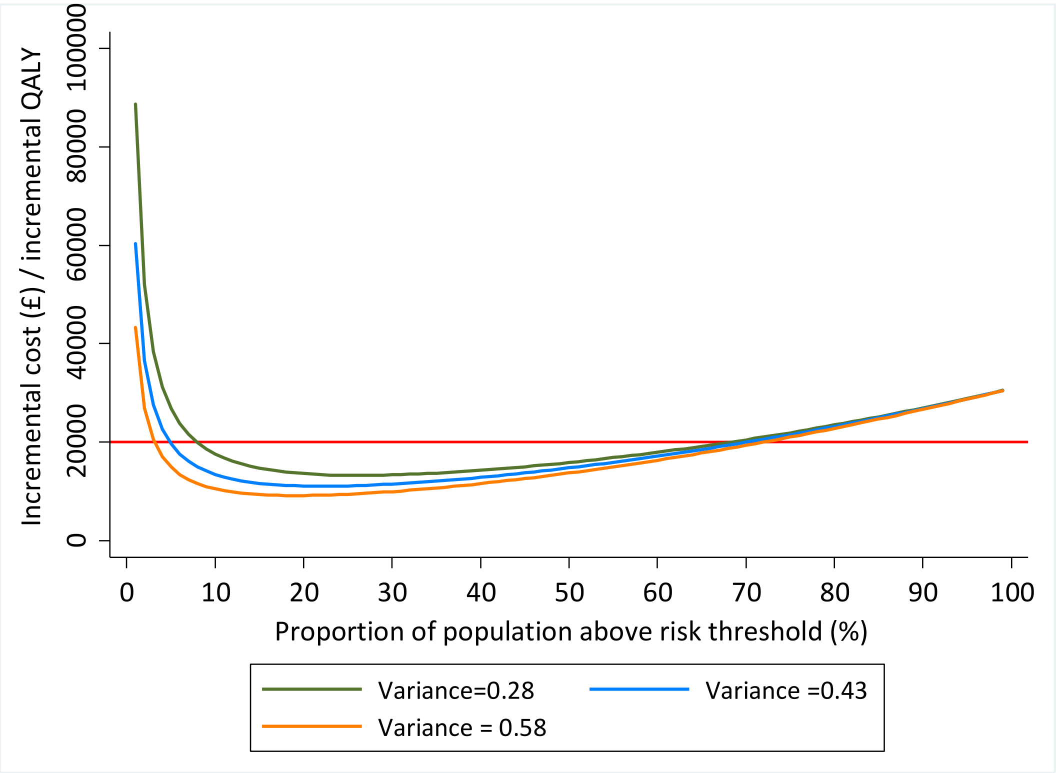

Red line: Threshold of £20,000 per QALY gained

In univariate deterministic sensitivity analyses, the baseline incidence of breast cancer (assuming screening advances the diagnosis by 5 and 9 years among women 50-69 years of age), adherence to screening recommendation (75% and 90%), cost of risk assessment (£25, £75 and £100) were varied. In each case, with higher baseline incidence, lower adherence, and higher cost of risk assessment, the ICER would fall below £20,000/QALY at higher percentile of risk threshold. Varying the variance of the risk distribution (0.28 and 0.58) did not materially affected the result.

The ICER, WTP, and what is deemed acceptable benefit-harm trade off will vary across healthcare systems. However, the different sensitivity analyses indicate that risk-based screening improves the cost-effectiveness of the screening programme – this trend is likely to apply to other healthcare systems, though the optimal risk threshold for targeted screening would vary across healthcare systems.

© 2018 Pashayan N et al. *JAMA Oncology.*

eReferences

1. Pharoah PD, Sewell B, Fitzsimmons D, Bennett HS, Pashayan N. Cost effectiveness of the NHS breast screening programme: life table model. *BMJ* 2013; 346:f2618.
2. The Independent UK Panel on Breast Cancer Screening. The benefits and harms of breast cancer screening: an independent review. *The Lancet* 2012; 380(9855):1778-1786.
3. Office of National Statistics. Reference table: Population estimates for England and Wales, mid-2002 to mid-2010 revised (National). 2012. Available: http://www.ons.gov.uk/ons/publications/re-reference-tables.html?edition=tcm%3A77-269171 Accessed December 18, 2017.
4. Clarke M, Collins R, Darby S, Davies C, Elphinstone P, Evans V et al. Effects of radiotherapy and of differences in the extent of surgery for early breast cancer on local recurrence and 15-year survival: an overview of the randomised trials. *Lancet* 2005; 366(9503):2087-2106.
5. Ara R, Brazier JE. Using health state utility values from the general population to approximate baselines in decision analytic models when condition-specific data are not available. *Value Health* 2011; 14(4):539-545.
6. Peasgood T, Ward SE, Brazier J. Health-state utility values in breast cancer. *Expert Rev Pharmacoecon Outcomes Res* 2010; 10(5):553-566.
7. Breast screening programme. NHS, 2017. Available: http://webarchive.nationalarchives.gov.uk/20150506050724/http://www.cancerscreening.nhs.uk//breastscreen/cost.htm . Accessed December 18, 2017.
8. Department of Health. NHS rereference costs 2012 to 2013. 2015 Available: https://www.gov.uk/government/publications/nhs-reference-costs-2012-to-2013. Accessed December 18, 2017.
9. National Institute for Health and Care Excellence. Early and locally advanced breast cancer: diagnosis and treatment. Clinical guideline [CG80]. 2017. Available: https://www.nice.org.uk/guidance/cg80 . Accessed December 18, 2017.

© 2018 Pashayan N et al. *JAMA Oncology.*
